# Critical reflection on required service to the community propels prospective medical students toward higher empathy, compassion, and bias mitigation but are these gains sustainable?

**DOI:** 10.3389/fmed.2022.976863

**Published:** 2022-09-09

**Authors:** Lon J. Van Winkle, Bradley O. Thornock, Brian D. Schwartz, Alexis Horst, Jensen A. Fisher, Nicole Michels

**Affiliations:** ^1^Department of Medical Humanities, Rocky Vista University, Parker, CO, United States; ^2^Department of Biochemistry, Midwestern University, Downers Grove, IL, United States; ^3^Department of Medical Humanities, Rocky Vista University, Ivins, UT, United States

**Keywords:** bias mitigation, cognitive empathy, compassion, critical reflection, dissonance reconciliation, healthcare curricula, self-examination, service-learning

## Abstract

**Purpose:**

We observed increased cognitive empathy and reflective capacity scores when prospective medical students wrote critical reflections on mandatory team service-learning in a Medical Humanities course, but these findings did not include a control group. Here we compare these survey results in similar courses with and without required service-learning.

**Methods:**

Forty-three prospective medical students completed a Medical Humanities course requiring critical reflection on team service-learning. In comparison, 32 students finished a similar course in which service to the community was not mandatory. Before starting the courses, students completed reliable surveys of their cognitive empathy and reflective capacity, and more than 93% of the students completed the same surveys after finishing the courses.

**Results:**

Students' cognitive empathy and reflective capacity scores increased significantly when service-learning was required, but the scores did not increase significantly when service to the community was not required. The effect size for the empathy increase was of crucial practical importance (*r* = 0.50), whereas it was of moderate practical importance for the increase in reflective capacity (*r* = 0.34).

**Conclusions:**

These and prior findings strongly support the conclusion that students' critical reflection on mandatory team service-learning fosters development of their cognitive empathy and reflective capacity. We present a model program to incorporate critical reflection on service to the community throughout the curricula of all healthcare professions trainees.

## Introduction

Compassionate care fosters better patient outcomes in healthcare settings, and cognitive empathy initiates this compassion (1). Nevertheless, “compassion fatigue” plagues roughly half of healthcare practitioners (2). Consequently, healthcare professions students witness inconsistent preceptor empathy and models of compassionate behavior especially when they enter clinical training (3). As a possible solution to this shortcoming, structured cognitive empathy and compassion training could be added to the curricula of all health professions students (1), but how might such andragogy be implemented and maintained?

We Van Winkle et al. (4, 5) and others (6) have argued forcefully for using evidence- and team-based service-learning to foster and maintain high levels of cognitive empathy and compassion in healthcare professional students. The benefits to students' professional development are enhanced even further when they write critical reflections on their service-learning experiences (7–10). Such reflection on service to the community should continue in association with team-based learning throughout trainees' careers as students and, less formally, into their professional lives (4, 5).

For example, we have found that medical students' patient-centered orientations are enhanced in association with their written team-based critical reflections on the challenges of medicine and medical education, but this greater caring about patients persists in students only so long as they continue to formally reflect (11). Similarly, medical students' cognitive empathy scores increased when they wrote such reflections on service-learning experiences, but higher empathy scores were maintained only as long as they continued their team service to the community (12).

Service-learning elicits dissonance on which to reflect partly by exposing implicit biases held by students (13). Such biases adversely affect patient care and outcomes (14), and their mitigation through written critical reflection appears to enhance cognitive empathy and compassion (13, 15, 16). Our main purpose in this study was to test whether service-learning is essential to produce and maintain increases in cognitive empathy scores among prospective medical students. Versus whether reflective writing on the challenges of medical practice, medical education, and other humanitarian considerations are sufficient to produce gains in empathy and compassion. We also wanted to test whether increases in students' reflective capacities may be partly responsible for their increases in empathy.

Hypothesis 1: Jefferson Scale of Empathy (JSE) (17) and Reflective Capacity (RC) (18) scores will increase in prospective medical students in association with a Medical Humanities course requiring written critical reflections on team service-learning, but such will not be the case when service-learning is not required of teams.
Hypothesis 2: The known correlations between JSE and RC scores in prospective medical students can partially account for increases in these scores in association with a Medical Humanities course requiring written critical reflection on service-learning.

## Methods

### Participants and team formation

Prospective medical (Master of Science in Biological Sciences, MSBS) students at Rocky Vista University participated in this study from August to December 2021. Of these prospective medical students, 44 enrolled in the program in Parker, Colorado (CO), and 34 matriculated in Ivins, Utah (UT). One student in CO and two in UT withdrew from the program before the end of the semester. Of the remaining students in CO, 31 (72%) identified as female and 12 (28%) as male. Also, 28 (65%) identified as White, 6 (14%) as Asian, 4 (9%) as Black/African American, 3 (7%) as Hispanic, 1 (2%) as two or more, and 1 (2%) undisclosed. The ages of students in CO ranged from 20 to 40 years (mean = 25.5 years). Of the 32 students in UT, 17 (53%) identified as female and 15 (47%) as male. In addition, 19 (59%) were White, 1 (3%) Asian, 1 (3%) Black/African American, 8 (25%) Hispanic, 1 (3%) two or more, and 2 (6%) undisclosed. The ages of students in UT ranged from 21 to 35 years (mean = 25 years). In the CO section of the Medical Humanities course, teams of six or seven were formed randomly on the first day of class. Teams of five (in one case four) were formed randomly in the UT section of the course before the first day of Medical Humanities, because the teams also participated in team-based learning (TBL) in another course of the MSBS program. While teams in CO also worked together on projects and workshops in other courses, they did not participate in another formal TBL course.

Nevertheless, students in CO and UT are enrolled in the same MSBS program at Rocky Vista University. Consequently, students on the CO and UT campuses are required to have similar experiences. The student learning outcomes must be the same or very similar (as listed below for the two sections of the Medical Humanities course). The student learning outcomes for other courses in the program are identical as are the content of those courses in CO and UT. In fact, many of the courses are taught across campuses *via* Zoom. Hence, most examinations in other courses are identical or nearly identical as they cover the same material presented on both campuses.

### Medical humanities course descriptions

Student learning outcomes for the Medical Humanities courses were the same (outcomes 1, 3) or similar (outcomes 2, 4) in CO and UT and were as follows.

1. Discuss the major issues in professional identity formation and development particularly as they relate to relationship-centered learning and care.
2a. Demonstrate the benefits of collaborative, interdisciplinary learning through discussion, thorough consideration of diverse perspectives, service-learning, and written critical reflection. (CO)
2b. Demonstrate the benefits of collaborative, interdisciplinary learning through discussion, thorough consideration of diverse perspectives, and written critical reflection. (UT)
3. Focus in more profound ways on listening and communicating.
4a. Integrate knowledge from all courses with service-learning and the practice of medicine. (CO)
4b. Integrate knowledge from all courses with reflective writing and the practice of medicine. (UT)

In both CO and UT, 52% of students' grades were based on the level of critical reflection they exhibited in four reflective writing assignments (13, 15, 16). In CO, however, major focuses of the writing were on experiences in planning and performing team service-learning, whereas service-learning was not expected or required in UT. In CO, students' reflective writing assignments were assessed by one of us (LV) for dissonance, dissonance reconciliation, self-examination, and critical reflection (CR), as defined and reported previously (13). By this definition, “self-examination (including CR) was exhibited when a student recognized how their thoughts (and behaviors) did not match their personal and humanistic values; experienced perplexity, doubt, hesitation, or mental difficulties (i.e., dissonance); and began to decide how better to align their thoughts (and behaviors) with their values (i.e., dissonance reconciliation).” That is, both dissonance and dissonance reconciliation had to occur for self-examination and CR to be present.

Otherwise, individual and team quizzes over reading assignments in a TBL format were the same in both CO and UT for three-fourths of these readings. These assignments included “If I Understood You Would I Have This Look on My Face?” by Alan Alda (19), Chapter 3 of “Narrative Medicine” by Rita Charon (20), and “What Patients Say, What Doctors Hear” by Danielle Ofri (21). Similarly, team and class discussions of the readings had the same format in both CO and UT. Also, students in both CO and UT completed several Implicit Association Tests. In the one-fourth of the courses for which readings differed, students in CO read “The Empathy Effect” by Helen Riess (22), while UT students read “Klara and the Sun” by Kazuo Ishiguro (23). Students in both UT and CO also watched a 50-min segment of a Gates Foundation video concerning bias.

### Data collection

Students on both campuses completed online versions of the Jefferson Scale of Empathy (JSE) (17) and the Reflective Practice Questionnaire (RPQ) (18, 24) before beginning the course in August and after finishing the course in December (13, 15, 16). The 40-item RPQ measures reflective capacity (RC) and six related characteristics (18, 24). Cronbach's alpha values for the seven subscales have ranged from 0.75 to 0.91 (18, 24), while the values of subscales presented under results for our students were 0.85 and 0.90 for general confidence (GC) and reflective capacity (RC), respectively. See Rogers et al. (18) online for a current version of the RPQ.

Students completed the 20-item JSE with permission of the copyright holder (© Thomas Jefferson University, 2001, all rights reserved). The JSE, HPS-Version, has had a Cronbach's alpha value of approximately 0.84 in numerous studies [e.g., (17, 25)], while this value was an acceptable 0.79 for the present study. This somewhat lower value may reflect our smaller student sample size than for most studies presented in the literature. See a current version of the scale online in Fjortoft et al. (25). According to numerous studies, the JSE reliably measures healthcare professions practitioners' and students' cognitive empathy [reviewed in (17)].

Finally, CO students completed an in-house paper survey on the last day of class in December 2021 concerning their feelings about team-based learning, implicit bias, and service to the community (13, 15, 16). While UT students did not complete this survey, they did write team reflective papers on these topics.

An administrative assistant collected surveys and used random numbers to pair students' RPQ and JSE responses in August to those in December. To maintain anonymity, the administrative assistant destroyed all records associating students with their survey responses. That is, the data were retained, but no survey could be connected to the student who completed it. In CO, survey response rates in December were 100, 98, and 93% for the in-house, RPQ, and JSE, respectively. In UT, the response rates were 94% in December for both the JSE and RPQ.

The Rocky Vista University Institutional Review Board (IRB) reviewed this study (HIRB# 2018-0006) and determined that it satisfies the criteria for exemption. Students gave written informed consent to publish excerpts from their written critical reflections.

### Statistical analyses

Students' JSE, RC, and General Confidence (GC) scores in December were compared to their scores in August using paired *t*-tests. JSE, RC, and GC scores in CO were compared to those in UT using unpaired *t*-tests. One-sample *t*-tests were used to determine whether responses to in-house survey items were significantly different from neutral. During *t*-tests, effect sizes (*r* values) were also calculated. ROUT (Q = 1%) was used to detect values that were outliers in each data set. GraphPad Prism 8.3.0 Software Inc. (La Jolla, CA) was used for all statistical analyses including two-way analysis of variance (ANOVA) for Cronbach's alpha calculations.

## Results

### Value of service-learning

In CO, students' responses to items 1, 3, 5, 7, 9, and 10 in Table 1 each indicate a favorable impact of service-learning on students' professional development (significantly different than neutral, r = 0.86–0.96, *p* < 0.0001). In particular, every student agreed with the statement “Encounters with people/venues in our service-learning project helped me to see my potential biases toward people/venues more clearly” (item 10 in Table 1, significantly greater than neutral, *r* = 0.96, *p* < 0.0001). As in our prior studies (13, 15, 16), critical reflection on service to the community contributed significantly to students' bias mitigation not only as indicated by item 10 responses, but also according to the content of their written critical reflections (summarized below) and identification of their specific biases in response to the question “Of what biases did you become aware during encounters with people/venues in your service-learning project?” (Table 2).

**Table 1:** Medians and distributions of responses to questions regarding team- and service-learning were each significantly different from neutral (i.e., 4.0, *N* = 43, *p* < 0.0001, question 8 *p* = 0.0013).

**1**	**2**	**3**	**4**	**5**	**6**	**7**
**Strongly Disagree**	**Disagree**	**Somewhat Disagree**	**Neither Agree/Disagree**	**Somewhat Agree**	**Agree**	**Strongly Agree**
						**Median**
1. Having a team service-learning project in Medical Humanities was very engaging.						***7.0***
0	0	0	1 (2.3%)	3 (7.0%)	12 (27.9%)	27 (62.80%)
2. I would have been better off on another team in Medical Humanities.						***1.0***
33 (76.7%)	8 (18.6%)^*^	2 (4.7%)^*^	0	0	0	0
3. Next year, Medical Humanities should continue to expect teams of MSBS students to perform service-learning projects and to write reflections on their experiences with the projects.						***7.0***
0	0	1 (2.3%)^*^	1 (2.3%)	1 (2.3%)	12 (27.9%)	28 (65.1%)
4. All things considered, I could not have been assigned to a stronger team in Medical Humanities.						***7.0***
0	1 (2.3%)^*^	0	1 (2.3%)	2 (4.7%)	15 (34.9%)	24 (55.8%)
5. I gained very little from our service-learning project and written reflections on the project.						***1.0***
27 (62.8%)	11 (25.6%)	2 (4.7%)	2 (4.7%)	1 (2.3%)^*^	0	0
6. Medical Humanities should continue to use team-based learning in future courses.						***7.0***
0	0	0	3 (7.0%)	0	14 (32.6%)	26 (60.5%)
7. Writing reflections on our service-learning project fostered my professional development.						***6.0***
0	0	3 (7.0%)	3 (7.0%)	6 (14.0%)	17 (39.5%)	14 (32.6%)
8. Encounters with people in our service-learning project caused me to study for all of my courses with more interest than likely would have occurred without the project.						***5.0***
1 (2.3%)	2 (4.7%)	5 (11.6%)	11 (25.6%)	8 (18.6%)	9 (20.9%)	7 (16.3%)
9. Encounters with people in our service-learning project will help me to be engaged with people regardless of the setting or disposition of the person.						***7.0***
0	1 (2.3%)^*^	0	1 (2.3%)	3 (7.0%)	13 (30.2%)	25 (58.1%)
10. Encounters with people/venues in our service-learning project helped me to see my potential biases toward people/venues more clearly.						***7.0***
0	0	0	0	5 (11.6%)	16 (37.2%)	22 (51.2%)
11. Unconscious bias might affect some of my clinical decisions or behaviors as a healthcare professional.						***6.0***
0	1 (2.3%)	2 (4.7%)	2 (4.7%)	12 (27.9%)	13 (30.2%)	13 (30.2%)

* Significant outliers. The bold values are medians.

**Table 2:** Summary of written statements of biases expressed by students in the survey about their team- and service-learning experiences (40 of 43 students stated one or more of their biases in response to the question “Of what biases did you become aware during encounters with people/venues in your service-learning project?”).

**Nature of negative bias**	**Number of times expressed**
Substance abuse/addiction	10
Race/language	8
Gender	7
Sexual orientation	4
Obesity	3
Economic class/homelessness	3
Disabled/disabilities	3
Favor same as me	3
Awareness of implicit bias	3
Less educated	2
Age	1
Appearance/dress	1
Rude people	1
Religion	1
Wealthy people	1
Not caring for self	1
Working during COVID	1
Unequal access to healthcare	1
None	1

### Changes in JSE, GC, and RC scores with and without reflection on required team service-learning

**Figure 1:**
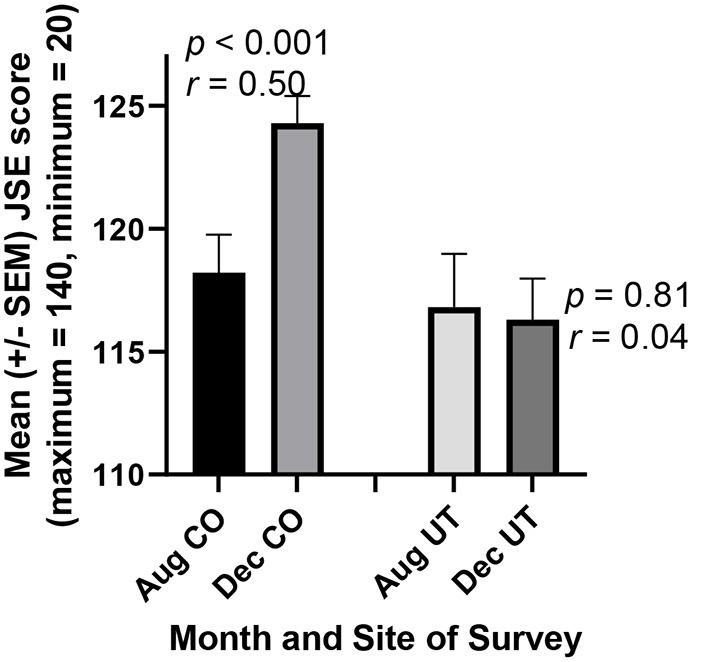
Prospective medical students Jefferson Scale of Empathy (JSE) scores increased in association with a Medical Humanities course requiring reflection on a team service-learning project (CO). But no increase was observed in an otherwise similar course where service to the community was not required (UT). In Colorado (CO), 40 of 43 students completed the scale both before the course began in August and after its completion in December, so their scores could be paired for more powerful statistical analysis (paired *t*-test). Similarly, 30 of 32 students in Utah (UT) completed the scale both prior to and following the course.

**Figure 2:**
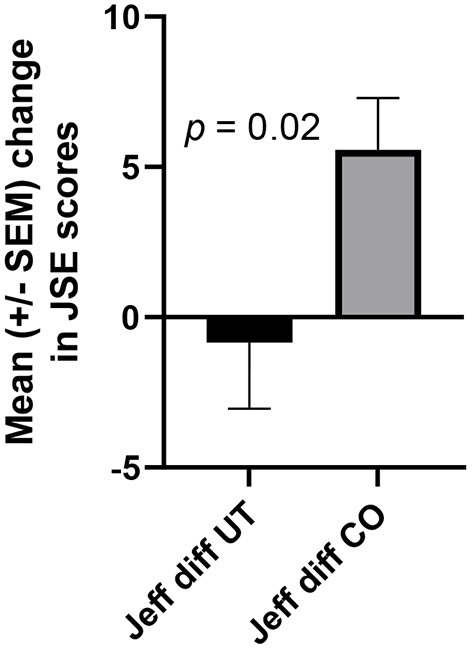
The changes in students JSE scores in CO significantly exceeded those in UT. Each student's score in August was subtracted from their score in December, and the resultant series of differences (diff) in CO (*n* = 40) and UT (*n* = 30) were compared statistically using an unpaired *t*-test.

JSE scores rose dramatically in association with written reflections on mandatory team service-learning (r = 0.50, *p* < 0.001), but no such increase occurred when service-learning was not required (Figure 1). As expected, when the change in each student's JSE score was calculated, the means of these score changes were significantly different and larger among students performing reflections on mandatory team service-learning than in students not performing required service to the community (Figure 2, *p* < 0.02).

**Figure 3:**
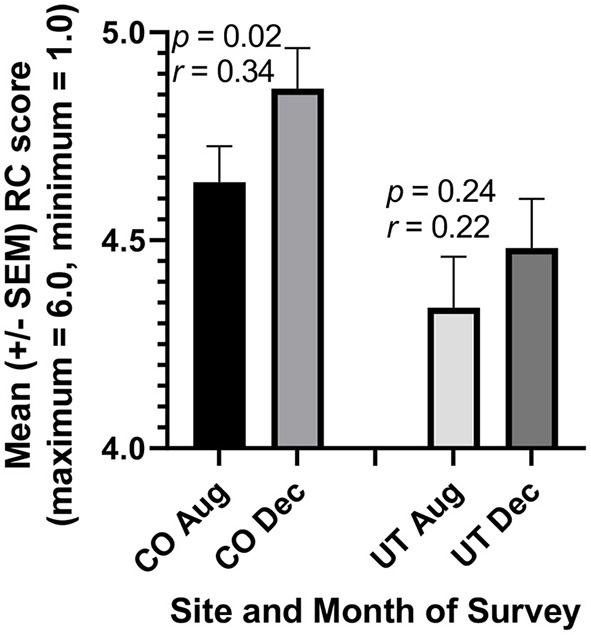
Prospective medical students Reflective Capacity (RC) scores increased in association with a Medical Humanities course requiring reflection on a team service-learning project (CO). But no increase was observed in an otherwise similar course where service to the community was not required (UT). In Colorado (CO), 42 of 43 students completed the RC subcomponent of the reflective practice questionnaire (RPQ) both before the courses began in August and after its completion in December, so their scores could be paired for more powerful statistical analysis (paired *t*-test). Similarly, 30 of 32 students in Utah (UT) completed this subcomponent both prior to and following the course.

Similarly, students' RC scores increased significantly in CO in association with written critical reflection on team service-learning (Figure 3, *r* = 0.34, *p* < 0.02), as was the case in our prior studies (13, 15, 16). No statistically significant increase was found among students not required to perform service to the community in UT. While the rise in RC scores for CO students was smaller than in previous such cohorts (13, 15, 16), this smaller change is largely attributable to RC scores being higher in CO students in August than they had been previously.

In a prior publication (13), we stated that self-examination/critical reflection is exhibited when “one recognizes how their thoughts and behaviors do not match their personal and humanistic values, experiences perplexity, doubt, hesitation, or mental difficulties (i.e., dissonance), and decides how better to align their values, thoughts, and behaviors (i.e., dissonance reconciliation). Thus, both dissonance and its reconciliation must be present in written reflections to define them as “critical reflection.” According to this definition, students in CO exhibited critical reflection a median of four out of four opportunities to do so. Moreover, 40 of 43 CO students showed critical reflection at least once, and 36 of 43 displayed critical reflections two or more times.

**Figure 4:**
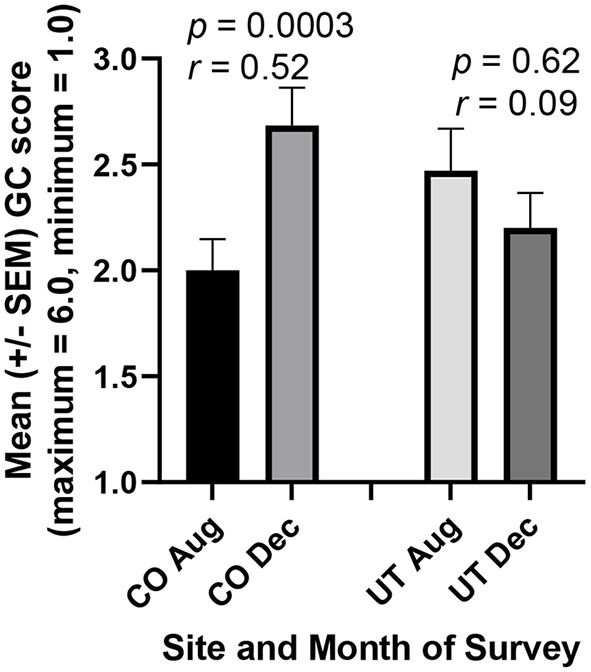
Prospective medical students General Confidence (GC) scores increased in association with a Medical Humanities course requiring reflection on a team service-learning project (CO). But no increase was observed in an otherwise similar course where service to the community was not required (UT). In Colorado (CO), 42 of 43 students completed the GC subcomponent of the reflective practice questionnaire (RPQ) both before the courses began in August and after its completion in December, so their scores could be paired for more powerful statistical analysis (paired *t*-test). Similarly, 30 of 32 students in Utah (UT) completed this subcomponent both prior to and following the course.

**Figure 5:**
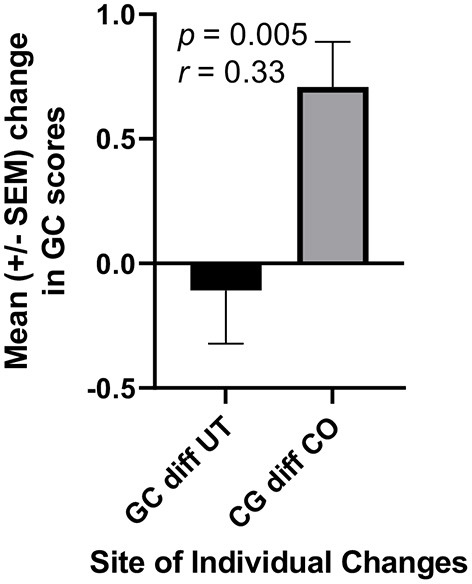
The changes in students GC scores in CO significantly exceeded those in UT. Each student's score in August was subtracted from their score in December, and the resultant series of differences (diff) in CO (*n* = 42) and UT (*n* = 30) were compared statistically using an unpaired *t*-test.

In addition to RC scores pertinent to hypothesis one, the RPQ collects data for six other dimensions. To avoid the statistical problem of assessing possible changes in several of these different subcomponents one-at-a-time, we first performed one-way ANOVA for these six dimensions together. Multiple comparison tests revealed a statistically significant difference only between the two sets of general confidence (GC) scores obtained for CO students in August and December (*p* < 0.01). GC scores increased significantly among students reflecting on required service-learning (*r* = 0.52, *p* = 0.003), but no increase was observed in students for whom service-learning was not mandatory (Figure 4). Also, when changes in individual student GC scores were calculated and compared between the UT and CO cohorts, the means of these individual changes were markedly different (Figure 5, *r* = 0.33, *p* = 0.005).

### Correlations between JSE and RC scores

We predicted previously that written critical reflection on required service-learning would be associated with an increase in JSE scores, since RC scores correlated significantly with JSE scores, and we had observed RC scores to increase in association with this mandatory activity (13, 15). As expected (13, 15, 16), RC scores correlated significantly with JSE scores in both CO and UT students in both August (CO, *r* = 0.32, *p* = 0.04; UT, *r* = 0.43, *p* = 0.01) and December (CO, *r* = 0.46, *p* = 0.003; UT, *r* = 0.60, *p* = 0.0005). However, the increases in RC and JSE scores among CO students appeared not to be interdependent.

**Figure 6:**
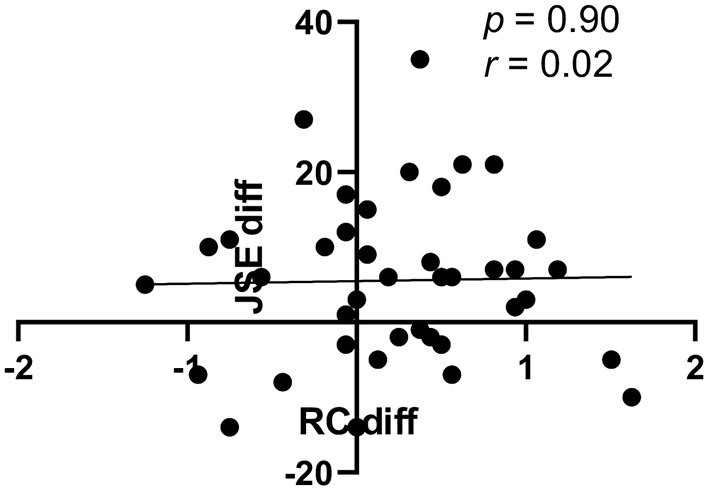
Correlation between individual changes in JSE and RC scores of students in CO. Each CO student's change in their JSE score between August and December is shown on the Y-axis (Jeff diff), while the change in the same student's RC score (RC diff) is plotted on the x-axis (*n* = 40).

**Figure 7:**
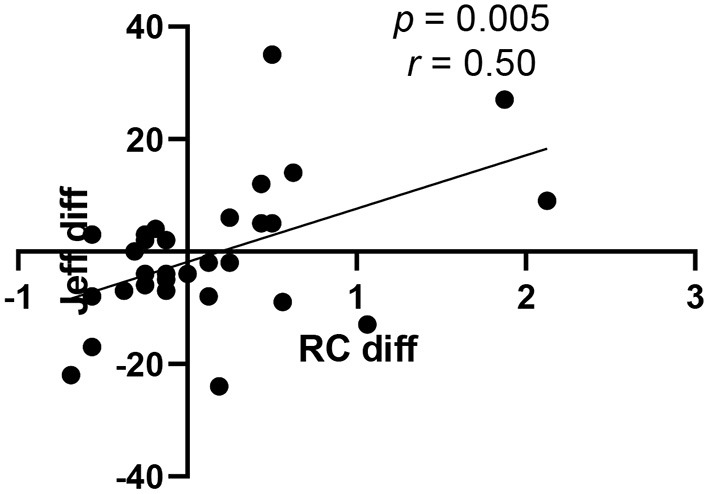
Correlation between individual changes in JSE and RC scores of students in UT. Each UT student's change in their JSE score between August and December is shown on the Y-axis (Jeff diff), while the change in the same student's RC score (RC diff) is plotted on the x-axis (*n* = 30).

When changes in individual JSE and RC scores between August and December were calculated for students in CO (e.g., Figure 2 for JSE scores), these differences in JSE and RC scores were not correlated (Figure 6, *r* = 0.02, *p* = 0.90). That is, students with greater increases in their JSE scores did not necessarily have the most improved RC scores. Somewhat surprisingly, however, these individual differences were correlated among UT students (Figure 7, *r* = 0.50, *p* = 0.005). Students in UT who improved their JSE scores also tended to increase their RC scores, while those who lost ground usually did so on both the JSE and the RC subcomponent of the RPQ.

### Team building, bias-mitigation, and compassionate behavior in CO students

Students' responses to items 2, 4, and 6 in Table 1 indicated students' affection for their teams and team-based learning. Especially regarding feelings for their teams, all students disagreed with the statement “I would have been better off on another team in Medical Humanities” (77% strongly so, item 2 in Table 1, significantly lower than neutral, *r* = 0.98, *p* < 0.0001). Also, all but two students agreed that they “could not have been assigned to a stronger team” (item 4 in Table 1, significantly greater than neutral, *r* = 0.93, *p* < 0.0001) even though only one team could have been the strongest. Interestingly, these attitudes developed although team memberships were assigned randomly, and many students expressed initially that they would have preferred to be on teams with friends they had already made. That is, students seemed to progress from biases against or neutrality toward their assigned teammates to strong biases in favor of them. In this way, they began to mitigate their biases. For example, one CO team reflected as follows at the beginning of the term following the Medical Humanities course.

“*…Although each week has come with its own “new schedule change,” thankfully our friendships that we established last (semester) have been consistent throughout. Within our last meeting, we chatted about how we've been and what has been hard for each of us, along with what we are excited about and our thoughts on returning to Food For Thought…”*

Similarly, CO students exhibited bias mitigation not only toward their teammates and in their written critical reflections (summarized above and below), but also in their responses in Table 1 (items 9, 10, and 11, each significantly greater than neutral, *r* = 0.81-0.96, *p* < 0.0001) and Table 2. Importantly, although three students denied that “unconscious bias might affect some of (their) clinical decisions or behaviors as a healthcare professional” (item 11 in Table 1), all three strongly agreed that encounters with people in their service-learning projects helped them to be engaged with people against whom they might have biases and that encounters with people/venues in their service-learning project helped them to see their potential biases toward people/venues more clearly (items 9 and 10 in Table 1). In a prior study, we found that such students plan to resist such biases as healthcare professionals (15).

Finally, regarding compassionate behavior, cognitive empathy is an important component of compassion (17), so the increase in JSE scores in CO students is quantitative evidence of their increasing compassion (Figures 1, 2). Qualitatively, we observed compassionate behavior by most students as reported in their written critical reflections in prior studies (13) and in the present one. For example, excerpts from three students' critical reflections were as follows:

“*…Initially, I was a little nervous to be jumping into a different opportunity so soon. I, personally, have never helped with a vaccine clinic before and being thrown into a COVID-19 vaccine clinic, with how important it is, was a little frightening. I didn't know what kind of people I would encounter; I wasn't familiar with the location of where the church resides, and I feared I would miscommunicate with patients due to possible language barriers. Thankfully, fears are all made within the mind, and they soon vanished when I arrived at the church…”*

“*…I was forced to address this anxiety as the vaccine clinic ran on and this actually helped me to make some level of peace with it. Starting off, I was clunky and awkward with my engagements and stumbled more than once with paperwork or how to address the people coming to my station. I couldn't speak Vietnamese to relay when to breathe in and out to help with the shot or discuss side effects that could come up from the booster. This started to improve, though, as the hours went on. The information that was more vital could still be acknowledged, from people helping to interpret for me or from me learning how to bypass it in other ways. Some of what I couldn't express in English I could describe as best as possible with body language. As a result, I started to relax as each wave of people came in and this made the experience more fun and made each interaction I had more meaningful…”*

“*… While I was very much looking forward to being a part of the goal to increase vaccination rates in the Denver area, I did have hesitancy - my job was to welcome in those who have arrived and review all of their information, but I don't know how to speak Vietnamese and I've never gone through paperwork for receiving a vaccination. I had to juggle passports, IDs, paperwork in Vietnamese and Covid histories, communicate with those that don't speak my language. I felt like I was making it harder for them to receive care because I don't speak their language and there could be many places where a miscommunication could lead to a mistake*.

*(However), there was a man who spoke Vietnamese and worked as an office coordinator for the physician supervising the pop-up clinic who helped me with the tough moments in communication. He told me that without the 7 of us there to volunteer, there would likely be many people who could not get vaccinated due to limited staffing…”*

### Team building, bias-mitigation, and compassionate behavior in UT students

Similar to CO, UT students also completed several assignments to mitigate bias, build team loyalty, and foster compassionate behavior. UT students were required to write three critical reflection papers—two individual reflections and one team reflection. For the two individual reflections, students were asked to reflect on a conflict or significant interaction they had with another person. For the first reflection, the students wrote about the conflict or interaction from their own perspective, focusing on the role their own values and biases played, and any dissonance between their preferred thoughts and behavior and their actual conduct. For the second reflection, students reconsidered the same conflict or interaction, but from the perspective of the other person involved. In this case, the students were instructed to charitably reflect on the possible values and biases of the other person, and how that other person might have viewed the student's behavior. As an exercise in fiction writing, many students reported that this second reflection was trying yet rewarding. For example, one student wrote:

“*The reflection allowed me to learn how biases can affect my interactions, especially in the second personal reflection, which required me to put myself in someone else's shoes and think about the situation. Writing from another person's perspective was exceedingly difficult, however it allowed me to gain a better understanding of how my own actions impact intersubjective spaces, especially when viewing it from another perspective.”*

In addition to writing fiction, UT students also read the novel *Klara and The Sun* by Kazuo Ishiguro. By reading fiction, it was hoped that the students would stretch their moral imagination and explore their own biases in situations outside of the norm. The novel touched on themes of grief, death, religion, genetic enhancement, and artificial intelligence. The fantastical world of the novel encouraged students to step beyond their initial or received biases and beliefs about the “real” world.

“*Klara and The Sun allowed for interesting discussions regarding ethics. Many times, our team was split on our views of what was ethical and what wasn't, but it was very refreshing to have conversations with different perspectives while continuing to create an inclusive environment. It also allowed us to really open up and put ourselves in character's shoes in regard to their views of care and grieving. Through these discussions we were able to learn about some of the biases we might have and how that impacts our views on patients.”*

Like the CO campus, the UT students worked in small teams for in-class discussions as well as for the team reflection paper. Every week, students discussed probing questions in their small teams and turned in minutes from their discussions. In addition, students met several times throughout the semester to work on the reflection assignments. During these meetings, each student took turns reading drafts of their individual reflections to their teammates, working together to finalize the assignments. The students also wrote a team reflection critically reflecting on the course as a whole—including the readings, in-class discussions, and earlier team meetings/reflections. Like the CO students, the UT students found this team work to be very beneficial.

“*In initial discussions, we all were worried about opening up about our experiences in fear of how we could be misconstrued or portrayed to each other because of the nature of the deep personal scenarios that we were introduced to. That was quickly set aside when we began opening up to each other about what it meant to be human and we learned that we were all very similar as we analyzed and grew from these social situations…We have also learned that being cognizant of others' emotions and experiences is largely a learned behavior that takes practice, and that successful physicians are able to hone in on their communication skills with patients despite some rifts in background and experiences.”*

As indicated below, their acquired ability to reflect may have prepared UT students to become more empathetic and compassionate in the following spring semester.

### Surprising results of follow up surveys

Completion of follow up surveys was requested of students at the end of the spring semester in April 2022. During the spring semester, written individual and team critical reflections on service-learning were required of UT as well as CO students in their Immunology course. This mandatory assignment was administered in the same way as in the CO Medical Humanities course during the fall semester of 2021.

As predicted based on our results (Figures 1–5) and prior studies (12, 13, 15, 16, 26), inclusion of critical reflection on required service-learning led to increases in UT students' JSE (*r* = 0.58, *p* = 0.01) and RC (*p* = 0.07) scores. While the students' optional response rate was only about 50% for the April 2022 UT surveys, the means of these JSE and RC scores for this sample of half the students in December were nearly identical to the means for the close to complete (94%) responses of students in December 2021 (Figures 1, 3).

Surprisingly, however, the gains in JSE and RC scores made by CO students during the fall semester of 2021 (Figures 1–3), were lost almost completely by the end of April 2022. This decrease in paired JSE scores in CO students between December and April was of crucial practical importance (*r* = 0.51, *p* < 0.01). Similarly, RC scores decreased in CO students between December and April (*r* = 0.49, *p* = 0.016). Nevertheless, CO students continued to evaluate their thoughts and behaviors in written critical reflections during the spring 2022 semester. Such assessments of oneself increases skills such as for general confidence (27) the greater scores of which in December (Figure 4) were maintained in CO students during the spring semester (*r* = 0.45, *p* = 0.025). While the CO students' optional response rate was only about 65% for the April 2022 CO surveys, the means of these JSE, RC, and GC scores for this sample of 2/3 of the students in December were nearly identical to the means for the close to complete (about 95%) responses of students in December 2021 (Figures 1, 3, 4).

## Discussion

Implicit bias remains a largely unmanaged issue in the US (28–30) and other countries (31, 32). The negative feelings of healthcare professionals against people of color causes discrimination against them. As a result, these patients experience poorer outcomes of their care. At another level, biases among healthcare professionals themselves can adversely influence patient care through such things as unwarranted discrimination against the otherwise most qualified applicants for residencies (33, 34). However, the methods used here to foster compassion through implicit bias mitigation in virtually every prospective CO medical student might be employed to help train all health professions students. This andragogy would improve not only the health of people of color, but also public health in general.

Implicit association tests helped our students mitigate biases against people for their color, body weight, gender, and sexual orientation. Then students' service-learning experiences expanded the list further to include people addicted to/abusing drugs, homeless people, and those who are disabled (Table 2). Thus, students learn to recognize new and unanticipated biases they need to mitigate in order not to discriminate in their delivery of healthcare. Furthermore, our students viewed the last portion of a Gates Foundation video showing healthcare workers and others around the world mitigating their implicit biases to avoid discriminating against their patients or other people they serve (View the video at: https://youtu.be/1SbUSj5iEgs).

Others have used a similar approach termed a pedagogy of discomfort (28, 35), and our methods and results resemble Sukhera and associates' transformative learning model (36). These authors foster disorienting experiences, such as exposing students' unconscious biases or requiring service to the community, to produce critical reflection and self-examination, as we do for prospective medical students (13, 15, 16). As outlined in Figure 1 of (36), their students then work to be more compassionate and learn new skills to help people they serve.

In the present study, required reflection on team service-learning was associated not only with bias mitigation, but also with increased cognitive empathy, reflective capacity, and general confidence. The JSE is a valid and reliable measure of cognitive empathy (17), while the validity of the reliable reflective capacity component of the RPQ is emerging (13, 15, 16, 18). In contrast, increases in these survey scores were not observed when service-learning was not required (Figures 1–5). Other factors, such as the different environments in CO and UT (other than service-learning), could help to account for increases in survey scores in one but not the other location. Nevertheless, our prior results in the humanities course at both locations (13, 15, 16), as well as results for medical and pharmacy students at another institution (12, 26), strongly support the conclusion that critical reflection specifically on service-learning fosters development of greater cognitive empathy and compassionate behavior in healthcare professions students. Moreover, numerous recent papers call for the development of sustainable programs to foster and maintain empathy and compassion in these students [e.g., (1, 14, 37)], and reflection on service-learning is known to accomplish such ends (6, 27).

### Model curricular component to foster and sustain empathy, compassion, and bias mitigation through critical reflection on service to the community

#### Description of the proposed curricular component

Curricula promoting empathy, compassion, bias mitigation, and concomitant professional development requires critical reflection on service-learning throughout healthcare professional students' careers. We suggest employing team support systems beginning at the start of undergraduate training using both in-person team meetings and virtual technology. While interprofessional teams are desirable, they are not required for critical reflection on service learning to succeed (4, 12, 38, 39). Nevertheless, many institutions have the resources to form teams of five to seven members from various healthcare professions. When teams are interprofessional, they can begin not only to address core interdisciplinary competencies, such as communication, but also reflect on their biases for or against their own as well as other healthcare professions.

In our experience, faculty facilitators are unnecessary for teams to function well (12, 13, 15, 16, 26), and team members should begin meeting as soon as possible after they matriculate. For curricula that also use team- or problem-based learning, team development can be facilitated further if the same team membership is maintained for these other activities (12, 26). At their initial team meetings, members should begin to get to know one another, consider possible team names, and work to select a service-learning project. Each student ought to perform at least 5 h of service to the community each academic term, and, ideally, team members should perform service together. At regular intervals, but at least four times per term, each student should prepare written critical reflections about the service-learning they are planning/performing and their thoughts and feelings about the service and people served. While not usually central to the content of the reflections, students should also consider how their service relates to the course content of their curriculum and other experiences in their lives.

Written critical reflections help students move past superficial course content-related learning and supports their non-cognitive development. Teams should discuss members' written reflections at team meetings and provide feed-back and their in-action reflections at the meeting and later after reflecting on their own. Written reflections often include challenging experiences which may also inspire, affirm, or awe (4, 39). New understanding and meaning evolves from student and team discussions and critical reflections (4, 5, 12, 13, 15, 16, 26).

At team meetings, members support one another as they discuss critical reflections. During each academic term, teams should hold at least four, 90-min, face-to-face discussions. In this way, teams of five to seven members can consider each other's written critical reflections. And they can reserve time in their final meeting to summarize their experiences and reflections for the term. In addition to more profound understanding of themselves owing to written reflections on their experiences, students may comprehend one another's professional expertise and responsibilities more deeply when students from more than one discipline are included in teams. The latter occurs when students choose to share their experiences as they proceed through training for their field. Teams should provide written meeting minutes and their responses to each other's written critical reflections when they collect and submit individuals' reflections. Team discussions can continue when students begin clinical experiences either in person if possible or through virtual technology when necessary.

Teams should work together for as long as possible to continuously benefit from mutual support. Depending on the extent to which some teams may have lost members, it might become beneficial for remaining members to join another small team. In this way, students can remain active in team reflection, support, and professional development throughout their program.

#### Use of the followup surveys to help envision more sophisticated, nuanced, and sustainable curricular components

In a follow-up to the present study, the novel experience of selecting and performing required team service-learning appeared to foster UT students' empathy during their second, spring semester. In their written reflections, many UT student teams expressed catharsis owing to service-learning as a break from their even more demanding academic load in the spring. In keeping with this observation, the only subcomponent of RC to increase significantly in UT students during spring semester was reflection in action (RiA, *r* = 0.57, *p* = 0.028). Similarly, RiA was the only RC subcomponent to increase significantly in CO students during the fall semester (*r* = 0.39, *p* = 0.012). Thus, the initial challenge of selecting and performing team service-learning projects appears to have led students to “think on their feet” when interacting with people they chose to serve. This conclusion also is supported for CO students by their answers to items 9-11 in Table 1 which focus on how they expect to engage with others.

In contrast to UT students, however, we speculate that academic stressors overwhelmed CO students, so they lost most of the gains in empathy and reflective capacity achieved during the fall semester (Figures 1–3). Anecdotally, faculty members aware of morale in the CO MSBS cohort observed breakdowns in students' relationships, trust, and academic performances during the spring that were much more profound than any that might have occurred in prior years or in UT students. For example, academic grades of CO students averaged about three percentage points lower than those of UT students in the spring semester, and such lower grades cause deep concern in students hoping to earn admission to medical school. Moreover, in prior years, relationships among members of the class continued to grow as team members reached out to other people in their class, as well as team members, for support. We suggest that for CO students in the present study, the academic and other stressors were simply too profound for the positive effects of reflecting on service-learning to counter the negative stressors. We speculate that self-compassion (40) may have decreased among CO students during the spring semester, and this diminution in self-compassion led to the loss of empathy (a component of compassion) we observed in these students during the spring.

In our view, a proper balance, between stressors of academic curricula and activities intended to help relieve the stress, such as reflection on required service to the community, need to be properly balanced to produce the most cognitive and non-cognitive professional growth and development among students. Moreover, we suggest that the experiences of selecting and performing team service-learning may lose novelty and effectiveness if performed too frequently and only by themselves in the curricula. Instead, we suggest that these curricula be more dynamic with recurring but not necessarily continuous expectations for community service. During terms or rotations when written reflection on team service-learning is not required, one or more other of numerous empathy- and compassion-enhancing activities could be employed [e.g., (1, 14, 17, 37–41)]. The important element is that activities, such as reflection on service to the community, continue throughout students' training and careers in order to help them maintain meaning in their work.

#### Curriculum assessment and improvement

The curriculum should be evaluated regularly in several complementary ways. First, the faculty should assess all written reflections to determine the extent to which they exhibit self-examination and compassionate behavior. Evidence of such critical reflection is shown by students when they “recognize how their thoughts and behaviors do not match their humanistic and professional values; experience perplexity, doubt, hesitation, or mental difficulties; and begin to decide how better to align their values, thoughts, and behaviors” (12, 42). Using this definition, scores assigned by different faculty members for the extent of critical reflection exhibited by students correlate well (*r* = 0.92) (43). Such scores should in our view, be used as part of the grades earned by students in all concurrent courses and/or rotations. When students' grades depend, in part, on the quality of their written critical reflections, they seek guidance from the faculty on how to improve them when their scores are lower than they desire. Moreover, when team and individual reflections are scored as one, teams seek our advice and work together to improve the scores assigned to the team and, thus, to each of its members. When supplying feedback, we recommend using a “yes and” approach (19) where we indicate how teams' good starts can be made even better (13, 15, 16).

We have found such efforts to be feasible and not excessively increase our own or students' workloads. Each of about 500 students enrolled in biochemistry courses during the same term wrote critical reflections on their team service-learning and other related experiences on four occasions, and one of us assessed each of these reflections as described above (12, 26). Moreover, the pharmacy and medical student teams involved met to discuss their service and reflections every 2 to 4 weeks. Scores teams received on these assessments contributed to students' overall grades in the biochemistry courses.

Reliable and validated surveys should also be used to evaluate students' professional development in the proposed curricula. For example, we used the Reflective Practice Questionnaire (subcomponents include reflective capacity, desire for improvement, general confidence, confidence communicating with patients, uncertainty, stress interacting with patients, and job satisfaction) and the Jefferson Scale of Empathy (factors include “perspective-taking” and “compassionate care”) in the present study (Figures 1–5). Other quantitative measures of reflection, such as the Groningen Reflection Ability Scale (44), might also be used to assess students' progress toward greater reflective capacity, while a number of reliable and valid methods to assess empathy are known (41). Also, the Jefferson Scale of Attitudes Toward Interprofessional Collaboration (45) can be used to measure changes in feelings about other healthcare professions especially for students working in interdisciplinary teams. Additional surveys include more than 64 reliable and valid instruments to measure collaboration on teams of health-care professions students (46). We have never failed to observe improvement of students' attitudes, abilities, or cognitive empathy according to one or more of these quantitative scales even when critical reflection on service-learning lasted for only one quarter (12, 26) or semester in one course (13, 15, 16) as in the present case (Figures 1–5; Tables 1, 2). In our view, these gains can be maintained and even increased further when the interventions continue throughout healthcare professional students' curricula.

Nevertheless, the faculty should monitor all of these assessments and study the curriculum and its delivery for possible revision if students' progress is not maintained. In addition, not every CO student in the present study appeared to benefit from their experiences in Medical Humanities. For example, four of 40 CO students had JSE and RC scores that both decreased rather than increased in association with critical reflection on service-learning in the course (Figure 6). Thus, individual students' survey scores could be used to identify students in extended curricular programs who might benefit from individual attention to their experiences and resultant reflections (18). For example, critical reflection on service-learning can lead to change including discomfort and uncertainty. Some students may then become reluctant to change and resist or reject this andragogy (27). In the spirit of counseling such students, it might become useful to discuss with them the stages of critical reflection, and a variety of descriptions of these stages is available (summarized in 27). Students may even need to learn to detach, temporarily, from their critical reflections when the reflections become overwhelming to them (27).

## Limitations

In the present study, only 43 CO and 32 UT MSBS students were compared to determine the effects of team service-learning reflections on several aspects of CO students' professional development. However, our new results are consistent with our previous findings for 144 prospective medical students in our Medical Humanities course (13, 15, 16). Nevertheless, our data from a single university are difficult to generalize to healthcare-related programs at other universities. Our findings have, however, now been replicated with five cohorts of MSBS students. Such reproducibility indicates that we would likely obtain similar results for other programs at Rocky Vista University.

Moreover, reflection on team service-learning fostered compassion in nearly 500 medical, pharmacy, and masters students during Biochemistry courses at another institution (9, 10). Hence, we propose that our methods would be successful at other universities. We encourage other healthcare professional educators to determine whether teams of their students can (1) raise psychological safety and trust with one another using shared provocative experiences and (2) utilize the meaning they make of these experiences to become more critically reflective and to mitigate their implicit biases.

## Conclusions

Unfortunately, cognitive empathy scores decline or remain unchanged in most healthcare professional students especially when they enter challenging clinical training (17). Preventing–or better yet–reversing this direction in which components of compassion change in students should be an andragogic goal of all pertinent curricula. Compassion of clinicians toward their patients strongly influences patient wellbeing with marked improvements in their comfort, satisfaction, and physical health (4–6, 13, 15, 16). Here we demonstrate that empathy and other components of compassion need not decline but rather increase in association with students' critical reflections on service-learning and related experiences. We propose that such efforts should continue throughout healthcare professional students' training by incorporating them into their curricula (40). Such extended and continuous evidence-based interventions would foster the wellbeing of student teams when they are most vulnerable to their programs' hidden curricula. Ultimately, students' wellbeing prevents their burnout and translates into improved patient and public health (4–6, 13, 15–17, 40, 41).

## Data availability statement

The original contributions presented in the study are included in the article/supplementary material, further inquiries can be directed to the corresponding author.

## Ethics statement

The studies involving human participants were reviewed and approved by the Rocky Vista University Institutional Review Board (IRB) reviewed this study (HIRB# 2018-0006) and determined that it satisfies the criteria for exemption. Students gave written informed consent to publish excerpts from their written critical reflections. The patients/participants provided their written informed consent to participate in this study.

## Author contributions

LV, BT, BS, AH, JF, and NM: conceptualization, writing–review and editing, and visualization. LV and BT: writing–original draft. All authors participated in the editorial improvement of the text and approved the final manuscript.

## Conflict of interest

The authors declare that the research was conducted in the absence of any commercial or financial relationships that could be construed as a potential conflict of interest.

## Publisher's note

All claims expressed in this article are solely those of the authors and do not necessarily represent those of their affiliated organizations, or those of the publisher, the editors and the reviewers. Any product that may be evaluated in this article, or claim that may be made by its manufacturer, is not guaranteed or endorsed by the publisher.
